# NIR-MFCO dataset: Near-infrared-based false-color images of post-consumer plastics at different material flow compositions and material flow presentations

**DOI:** 10.1016/j.dib.2023.109054

**Published:** 2023-03-14

**Authors:** Nils Kroell, Xiaozheng Chen, Abtin Maghmoumi, Julius Lorenzo, Matthias Schlaak, Christian Nordmann, Bastian Küppers, Eric Thor, Kathrin Greiff

**Affiliations:** a Department of Anthropogenic Material Cycles, RWTH Aachen University, Wuellnerstr. 2, Aachen D-52062, Germany; b STADLER Anlagenbau GmbH, Max-Planck-Str. 2, Altshausen D-88361, Germany

**Keywords:** Sensor-based material flow characterization, Lightweight packaging waste, Mechanical plastic recycling, Circular economy, Machine learning, Computer vision, NIR spectroscopy, Polymers

## Abstract

Determining mass-based material flow compositions (MFCOs) is crucial for assessing and optimizing the recycling of post-consumer plastics. Currently, MFCOs in plastic recycling are primarily determined through manual sorting analysis, but the use of inline near-infrared (NIR) sensors holds potential to automate the characterization process, paving the way for novel sensor-based material flow characterization (SBMC) applications. This data article aims to expedite SBMC research by providing NIR-based false-color images of plastic material flows with their corresponding MFCOs. The false-color images were created through the pixel-based classification of binary material mixtures using a hyperspectral imaging camera (EVK HELIOS NIR G2–320; 990 nm–1678 nm wavelength range) and the on-chip classification algorithm (CLASS 32). The resulting NIR-MFCO dataset includes *n* = 880 false-color images from three test series: (T1) high-density polyethylene (HDPE) and polyethylene terephthalate (PET) flakes, (T2a) post-consumer HDPE packaging and PET bottles, and (T2b) post-consumer HDPE packaging and beverage cartons for *n* = 11 different HDPE shares (0% - 50%) at four different material flow presentations (singled, monolayer, bulk height H1, bulk height H2). The dataset can be used, e.g., to train machine learning algorithms, evaluate the accuracy of inline SBMC applications, and deepen the understanding of segregation effects of anthropogenic material flows, thus further advancing SBMC research and enhancing post-consumer plastic recycling.

**Specifications Table**

**Table:** 

Subject	Environmental Engineering
Specific subject area	Sensor-based material flow characterization in mechanical recycling processes
Type of data	Images
How the data were acquired	1. Binary mixtures of (i) high-density polyethylene (HDPE) and polyethylene terephthalate (PET) flakes, (ii) post-consumer HDPE packaging and PET bottles, and (iii) HDPE packaging and beverage cartons were created with different HDPE contents. 2. The binary mixtures were repeatedly captured on a conveyor belt under different material flow presentations simulating different measurement situations of inline-sensor technology in sorting and processing plants. 3. A *HELIOS NIR G2–320* hyperspectral imaging camera (990 nm – 1678 nm wavelength range) from EVK Kerschhaggl GmbH (Raaba, Austria) was used to pixel-based classify the material flows into pre-defined material classes. The resulting false-color images were captured.
Data format	• Raw • Preprocessed (cropped, spatially calibrated, white space removed)
Description of data collection	False-color images were created using the on-chip CLASS32 classification algorithm from EVK Kerschhaggl GmbH (Raaba, Austria) and exported as bitmap files (“raw data”). Afterward, the bitmap files were preprocessed in Python to ensure a spatially equidistant data representation and exported uncompressed as PNG files to ensure interoperability (“preprocessed data”). Both, raw and preprocessed data are available within the NIR-MFCO dataset, along with the Python code for preprocessing.
Data source location	• Institution: Department of Anthropogenic Material Cycles • City: Aachen • Country: Germany
Data accessibility	Repository name: Zenodo Data identification number: 10.5281/zenodo.7638775 Direct URL to data: doi.org/10.5281/zenodo.7638775
Related research article	N. Kroell, X. Chen, B. Küppers, J. Lorenzo, A. Maghmoumi, M. Schlaak, E. Thor, C. Nordmann, K. Greiff, Near-infrared-based determination of mass-based material flow compositions in mechanical recycling of post-consumer plastics: Technical feasibility enables novel applications, Resources, Conservation and Recycling 191 (2023) 106873. https://doi.org/10.1016/j.resconrec.2023.106873. [1]

## Value of the Data

• Contains false-color images from a total of *n* = 880 experiments of monitoring plastic material flows using near-infrared-based inline sensor technology on conveyor belts in combination with known material flow compositions for each experiment.
• The dataset is intended for researchers investigating novel applications of inline-sensor technology for the optimization of (mechanical) recycling processes [2]. Machine learning and computer vision researchers can use this dataset to train and assess different (machine learning) algorithms for predicting (mass-based) material flow compositions [1].
• Machine learning and image processing algorithms can be trained and assessed on predicting material flow compositions from near-infrared-based false-color images [1]. Furthermore, this dataset enables researchers to assess the accuracy of near-infrared-based inline material flow characterization under different measurement situations and can help gaining a better understanding of segregation effects of anthropogenic material flows [2].
• Data was collected from three test series (T1: HDPE and PET plastic flakes, T2a: post-consumer HDPE packaging and PET bottles, T2b: post-consumer HDPE packaging and beverage cartons) to simulate inline sensor technology applications in processing (T1) and sorting plants (T2).
• False-color images of four different material flow presentations (singled, monolayer, bulk height H1, bulk height H2 [1]) are included to simulate different sensor measurement situations in mechanical sorting and processing plants [1].
• For each test series and material flow presentation, *n* = 11 different material flow compositions have been recorded (0%; 0.1%; 0.5%; 1%; 2.5%; 5%; 10%; 20%; 30%; 40%; and 50% HDPE). Each experiment was repeated *n* = 10 (T1) and *n* = 5 (T2) times such that measurement repeatability and influences of different particle orientations can be quantified.

## 1 Objective

To increase plastic recirculation [3], mechanical plastic recycling processes need to be assessed and optimized, which requires known material flow compositions (MFCOs) [1,^2^,4]. Today, MFCOs in mechanical recycling are primarily determined through manual sorting analysis, which is time- and cost-intensive and thus often conducted on an irregular basis. In the future, inline sensor-based material flow characterization (SBMC) methods [2] could automate material flow characterization and enable novel SBMC applications to enhance plastic recirculation [4], [5], [6].

Numerous studies have demonstrated that post-consumer plastics can be classified with > 99% accuracy using near-infrared (NIR) spectroscopy at pixel and particle level [2]. However, little research has been conducted so far at the material flow level, specifically on predicting MFCOs [2]. A particular barrier for SBMC research on the material flow level is the higher experimental effort for creating ground truth data since a material flow comprises hundreds to thousands of individual particles. The NIR-MFCO dataset aims at expediting SBMC research by lowering the experimental barrier at the material flow level through providing NIR-based false-color images of post-consumer plastics for different particle types, materials, and material flow presentations in combination with their corresponding MFCOs.

## 2 Data Description

### 2.1 Dataset Structure

#### 2.1.1 Folder Structure

**Fig. 1:**
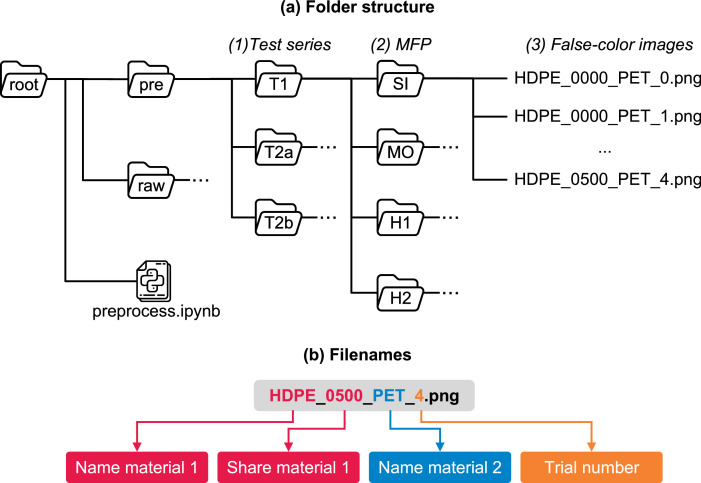
Overview of the NIR-MFCO dataset.

The NIR-MFCO dataset is constructed as a zip archive containing multiple levels of subfolders (Fig. 1a). In the first subfolder level, the full dataset is made available as *raw* (subfolder “raw”) and *preprocessed* (subfolder “pre”) data (cf. Section 3.4). Furthermore, we provide a Jupyter notebook to transform the raw into the preprocessed data (“preprocess.ipynb”).

At the second subfolder level, the dataset is structured in three individual **test series** (T1, T2a, T2b). Test series T1 contains false-color images of HDPE and PET plastic flakes at different material flow presentations (MFPs) and with different HDPE shares in volume percent (cf. Section 3.1.1). Test series T2a contains false-color images of post-consumer HDPE packaging and post-consumer PET bottles, while test series T2b contains false-color images of post-consumer HDPE packaging and post-consumer beverage cartons (BCs). The materials for T2 were sampled from a LWP sorting plant and HDPE shares are given in mass percent (cf. Section 3.1.2).

At the third subfolder level, the false-color images of each test series are structured into four different **MFPs** (SI, MO, H1, H2; cf. Section 3.2). Within each subfolder, false-color images of binary mixtures at *n* = 11 different HDPE shares are provided (cf. Table 1). Each experiment was repeated *n* = 10 (T1) and *n* = 5 (T2) times (each repetition is referred to as a *trial* in the following), resulting in *n* = 110 (T1) and *n* = 55 (T2a, T2b) false-color images per subfolder. Additionally, the third subfolder level of the *raw* dataset contains a folder “_calib” for spatially calibrating the false-color images (cf. Section 3.4.3). Table 1 summarizes the main characteristics of the NIR-MFCO dataset.

**Table 1:** Summary of the three test series included in the NIR-MFCO dataset.

Test series	T1	T2a	T2b
Test rig	lab	technical lab	technical lab
Material 1	HDPE	HDPE	HDPE
Material 2	PET	PET bottles	BC
Particle type	flakes	LWP articles	LWP articles
Particle size range	10 mm – 20 mm	60 mm – 240 mm	60 mm – 240 mm
Share unit	vol%	wt%	wt%
#Repetitions	10	5	5

MFPs	SI, MO, H1, H2		
Material 1 shares	0%; 0.1%; 0.5%; 1%; 2.5%; 5%; 10%; 20%; 30%; 40%; 50%		

#Images	440	220	220

#### 2.1.2 Format of Filenames

The material names of the binary mixtures (material 1 and material 2), material shares, and trial numbers are encoded within the filename as shown in Fig. 1b. In the filename, the share of a material is given directly after its name (cf. Fig. 1b). The material share is encoded as a dimensionless float (between 0 and 1) with three decimal place precision, and the comma point is not printed. For example, “0200” refers to a material share of 0.200, i.e., 20%; “0001” equals 0.1%, etc.

The share of the last material (material 2 for this dataset [$\emptyset_{\text{material}\;2}$]) is not given since it can directly be calculated from the other material shares (all material shares sum up to 100%), see Eq. (1).

(1) $$\emptyset_{\text{material}\;2}=1-\emptyset_{\text{material}\;1}$$

### 2.2 False-Color Images

#### 2.2.1 False-Colors

**Fig. 2:**
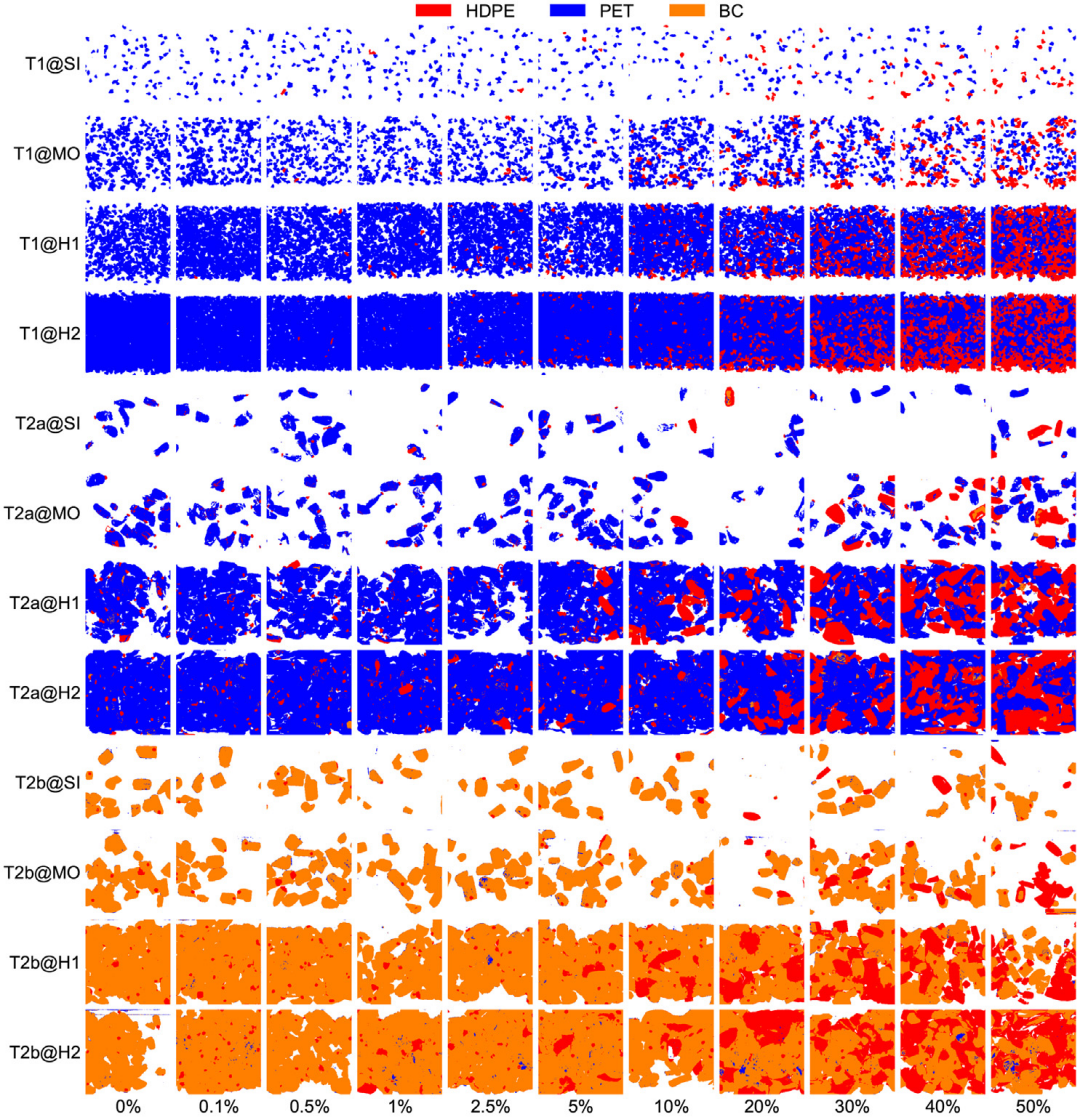
Exemplary false-color-image sections for test series T1, T2a, and T2b at different MFPs (rows) and HDPE shares (columns), cf. [1].

In each image, the pixel-based classification results from the near-infrared (NIR) sensor are represented as 8-bit RGB false-colors, as shown by the example image sections in Fig. 2. Table 2 summarizes the color values of all three classified materials (HDPE, PET, BC) and background color including their RGB values.

**Table 2:** Definition of false colors (8-bit red-green-blue [RGB] values) used for all three test series.

Material class	Color name	R	G	B
HDPE	red	255	0	0
PET	blue	0	0	255
BC	orange	255	127	0
Background	white	255	255	255

#### 2.2.2 Spatial Resolution

The raw false-color images of T1 have a spatial resolution of 1.64 mm/px in *x*-direction (conveyor direction, cf. Fig. 4) and 1.08 mm/px in *y*-direction (orthogonal to conveyor direction). For T2, the spatial resolutions are 3.98 mm/px and 3.50 mm/px in *x*- and *y*-direction, respectively. The preprocessed false-color images were spatially calibrated and thus have spatial resolutions of 1.08 mm/px in *x*- and *y*-direction for T1, and 3.50 mm/px in *x*- and *y*-direction for T2.

#### 2.2.3 Image Formats

The original false-color images from the NIR sensor are saved as 8-bit bitmap (“.bmp”) images (as provided by the EVK SQALAR software). For better interoperability, we save the preprocessed false-color images as 8-bit “.png” images.

## 3 Experimental Design, Materials and Methods

### 3.1 Materials

#### 3.1.1 Plastic Flakes (T1)

For test series T1, plastic flakes with a particle size between 10 mm and 20 mm were created to simulate typical SBMC applications in processing plants [7]. The plastic flakes were created using white high-density polyethylene (HDPE) and transparent polyethylene terephthalate (PET) plates with a thickness of 3 mm from S-POLYTEC GmbH (Goch, Germany).

First, the HDPE and PET plates were comminuted in a rotary shear (MOCO Maschinen- und Apparatebau GmbH & Co. KG AZ 7 [Viernheim, Germany]) with a peripheral speed of 0.5 m/s, cutting disk width of 28 mm, and drive power of 7.5 kW for pre-crushing. Second, the pre-crushed HDPE and PET particles were further comminuted in a cutting mill (RETO RECYCLINGTECHNIK GmbH GA 37/450 [Bergkamen, Germany]) with a peripheral speed of 9 m/s, rotor diameter of 350 mm, rotor length of 450 mm, output mesh size of 30 mm, and drive power of 37 kW.

Third, the plastic flakes were screened on an analytical sieve machine from Siebtechnik GmbH (Mühlheim [Ruhr], Germany) to produce plastic flakes in the desired size range of 10 mm to 20 mm, which is typically used in mechanical plastic recycling [7]. We used 10 mm and 20 mm round meshes and operated the screen at a speed of 1400 rpm for a sieving duration of 90 s.

**Fig. 3:**
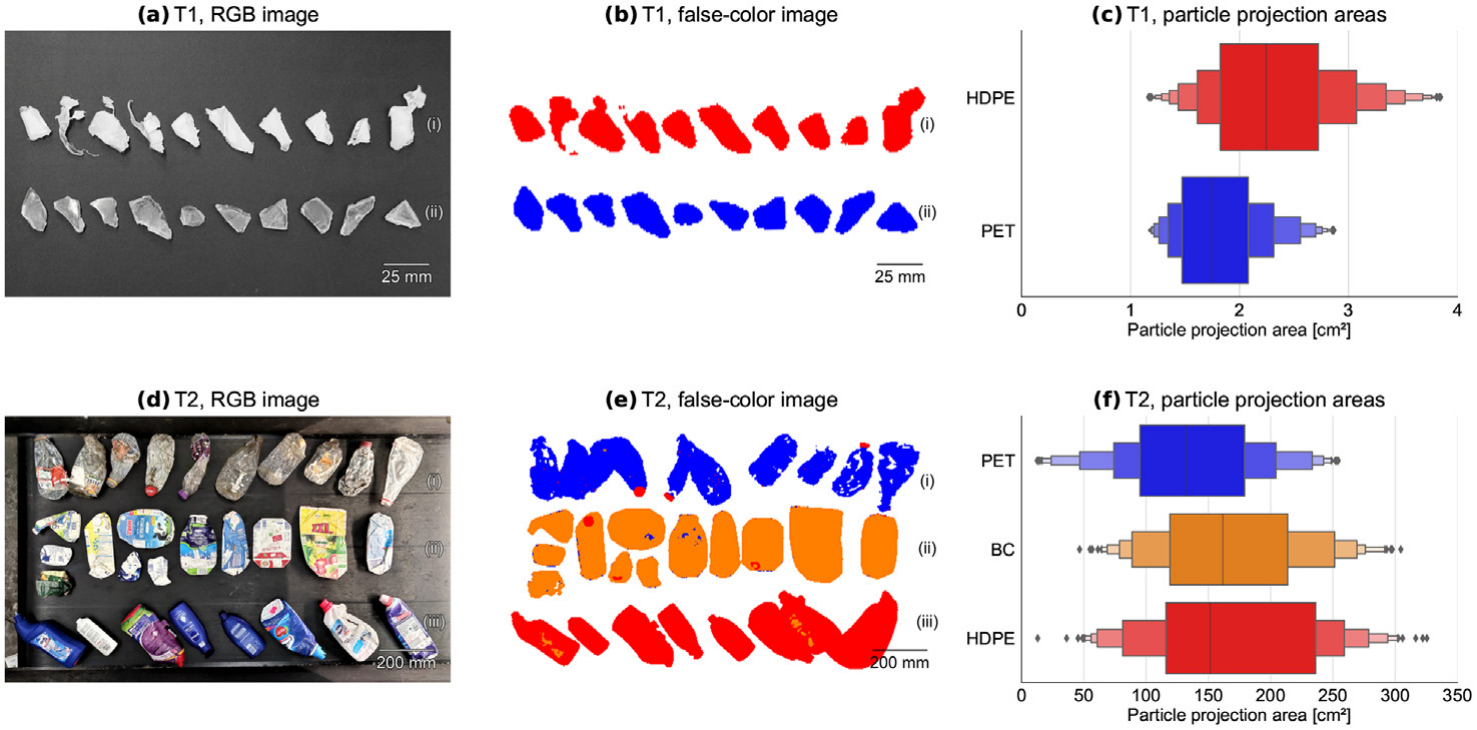
Materials. (a) RGB images and (b) NIR false-color images for test series T1 [(i) HDPE, (ii) PET], (d) RGB images and (e) NIR false-color images for test series T2 [(i) PET, (ii) BC, (iii) HDPE], (c, f) distribution of particle projection areas per material for test series T1 (plastic flakes) and T2 (post-consumer plastic packaging), respectively, cf. [1].

Fig. 3a-c shows exemplary RGB images (a) and false-color images (b), as well as projection area distributions (c) of the investigated plastic flakes. Grammages and raw densities of the investigated materials are summarized in Table 3.

**Table 3:** Grammages and raw densities of used materials within the NIR-MFCO dataset.

Test series	Material	Grammage [kg/m²]	Raw density [kg/m³]
T1	HDPE flakes	1.34^a^	960^b^
	PET flakes	2.66^a^	1270^b^

T2	HDPE packaging	2.46^a^	–
	PET bottles	2.30^a^	–
	BC	3.50^a^	–

Data source: ^a^determined based on bulk mass from balance and total projection area per bulk determined by NIR recordings, ^b^data sheet from S-POLYTEC GmbH (Goch, Germany).

#### 3.1.2 Lightweight Packaging (T2)

The goal of test series T2 is to simulate typical SBMC applications within sorting plants, in which material flows are presented as packaging articles [1]. Therefore, a sampling campaign was conducted in December 2020 at the LWP sorting plant Hündgen Entsorgungs GmbH & Co. KG in Swisttal (Germany). During the sampling campaign, each product fraction was sampled from the respective product fraction at the end of the technical sorting process and before manual sorting. From each product fraction (HDPE, PET bottles, and BC), a total volume of 1 m³ was sampled. To ensure maximum representativity during sampling, the full material flow was sampled from a continuously falling material stream according to LAGA PN98 [8]. The particle size range of the investigated LWP samples is approx. 60 mm – 240 mm [9]. Afterward, remaining impurities in the product fractions (fines [< 60 mm] and non-target material) were manually removed to obtain pure material fractions of each material. Fig. 3d–f shows exemplary RGB (d) and false-color images (e), as well as projection area distributions (f) of the investigated post-consumer plastic packaging. Grammages of the investigated materials are summarized in Table 3.

#### 3.1.3 Binary Mixtures

Three types of binary mixtures were generated to simulate the influence of different materials and particle types: (T1) HDPE and PET flakes, (T2a) post-consumer HDPE and PET packaging, and (T2b) post-consumer HDPE and BC packaging. For all three mixtures, *n* = 11 HDPE shares were investigated: 0%; 0.1%; 0.5%; 1%; 2.5%; 5%; 10%; 20%; 30%; 40%; and 50%.

To make the dataset easier transferable to mixtures with different density combinations (e.g., polypropylene, or polyvinyl chloride), HDPE shares for T1 are prepared in volume percent ($\varphi_{i}$ [vol%]). Since raw densities $\rho_{V}$ of PET and HDPE flakes are known (Table 3), mass- ($w_{i}$) and volume-based MFCOs ($\varphi_{i}$) can be converted into each other using Eq. (2).

(2) $$w_{i}=\;\frac{\varphi_{i}*\rho_{V,i}}{\sum_{j}\varphi_{j}*\rho_{j}}$$

For T2 (post-consumer packaging waste), an indication of material densities is not possible due to post-consumer waste characteristics (e.g., residual content, composites, hollow spaces). Therefore, material mixtures of T2 are created in mass percent ($w_{i}$ [*wt*%]).

### 3.2 Test Rigs

**Fig. 4:**
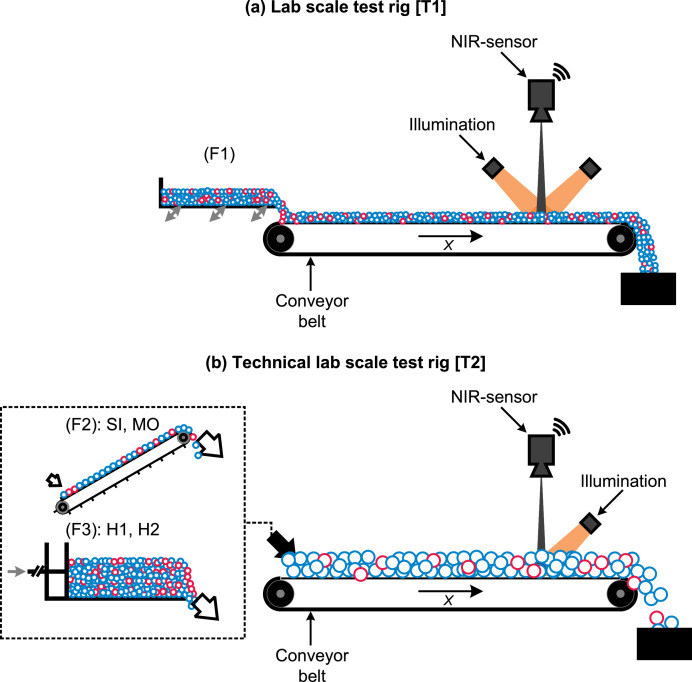
NIR test rigs for test series T1 (lab scale) and test series T2 (technical lab scale). Material feeding: (F1): vibrating conveyor, (F2): ascending conveyor, (F3): dosing bunker with stamp, cf. [1].

To simulate applications of inline sensor technology in processing and sorting plants, a lab-scale and technical lab-scale test rig were constructed. Each test rig consists of (i) a feeding unit and conveyor belt to create different MFPs and (ii) a NIR sensor for data acquisition (Fig. 4).

The created mixtures were presented in four different MFPs to the NIR sensor:

• Singled (**SI**): Particles do not overlap and (mostly) do not touch each other.
• Monolayer (**MO**): Particles (mostly) do not overlap but touch each other.
• Bulk height (**H1, H2)**: Particles overlap and touch each other and thus create a multilayered material bulk [1,2]. ($h_{2}>h_{1}$; $h_{1,T1}$ ≈ 10 mm, $h_{2,T1}$ ≈ 17 mm, $h_{1,T2}\;$≈ 150 mm, $h_{2,T2}$ ≈ 300 mm).

For T1, different MFPs were achieved through a vibrating conveyor (AViTEQ Vibrationstechnik GmbH KF 12–2 [Hattersheim am Main, Germany]) operated at different conveying speeds (Fig. 4a). For T2, an ascending conveyor (1 m/s conveying speed, 600 mm belt width, 25° ascending angle) was used for feeding in the SI and MO trials (Fig. 4b.F1), and a dosing bunker (1000 mm width; 850 mm height; 2000 mm length) with a stamp were used for the feeding in the H1 and H2 trials (Fig. 4b.F2). In both test series, black conveyor belts were used for material transportation (conveyor width: $b_{T1}$ = 385 mm, $b_{T2}$ = 845 mm; conveying speed: $v_{T1}$ = 0.25 m/s, $v_{T2}$ = 1 m/s).

Since the recording of the sensor data was technically limited to 60 s per trial (maximum recording time of uninterrupted false-color data), the material mixtures per trial were adapted to the respective MFP (Table 4). Each mixture was measured *n* = 10 (T1) and *n* = 5 (T2) times for each HDPE share and MFP, resulting in a total of *n* = 880 trials.

**Table 4:** Feed volume for individual test series and MFPs in liter [L].

MFP	T1	T2
SI	6	300
MO	10	300
H1	12	500
H2	12	500

### 3.3 Data Acquisition

#### 3.3.1 NIR Sensor

A *HELIOS NIR G2–320* hyperspectral imaging camera from EVK DI Kerschhaggl GmbH (Raaba, Austria) was used to capture and classify the NIR spectra in both test series. The used spectral range of the sensor was 990 nm to 1678 nm with a spectral resolution of 3.1 nm/band. The used NIR sensor has an on-chip classification engine, which is frequently used in different industrial and research applications [5,[10], [11], [12], [13], [14], [15], [16]. The resulting spatial resolution of the NIR sensor is 1.08 mm/px and 3.50 mm/px for T1 and T2, respectively (cf. Section 2.2.2). Four halogen lamps with a power of 400 W each were used as emitters and the reflection of radiation from the surface is captured by the NIR sensor. For test series T1, two halogen lamps each were illuminating the conveyor surface from front and back (Fig. 4a); for test series T2, four halogen lamps were illuminating the conveyor surface from front (against the conveyor direction) (Fig. 4b). Before each recording day, a black and white calibration of the NIR sensor was performed with the EVK SQALAR software using a white ceramic tile and switched on emitters (white calibration) and the black conveyor surface with switched off emitters (black calibration) [17].

#### 3.3.2 NIR Classification Model

For each test rig, a NIR classification model was developed to classify each spectrum into background (conveyor belt) and user-defined material classes (T1: HDPE, PET; T2: HDPE, PET, BC). For background definition, a threshold was defined to segment the recordings into background and foreground (materials) based on the mean intensity of each spectrum. For material classification, the on-sensor CLASS32 algorithm from EVK DI Kerschhaggl GmbH (Raaba, Austria) was used. In CLASS32, NIR spectra are first preprocessed (first derivative, normalization, and smoothing) and then compared to user-defined reference spectra.

**Fig. 5:**
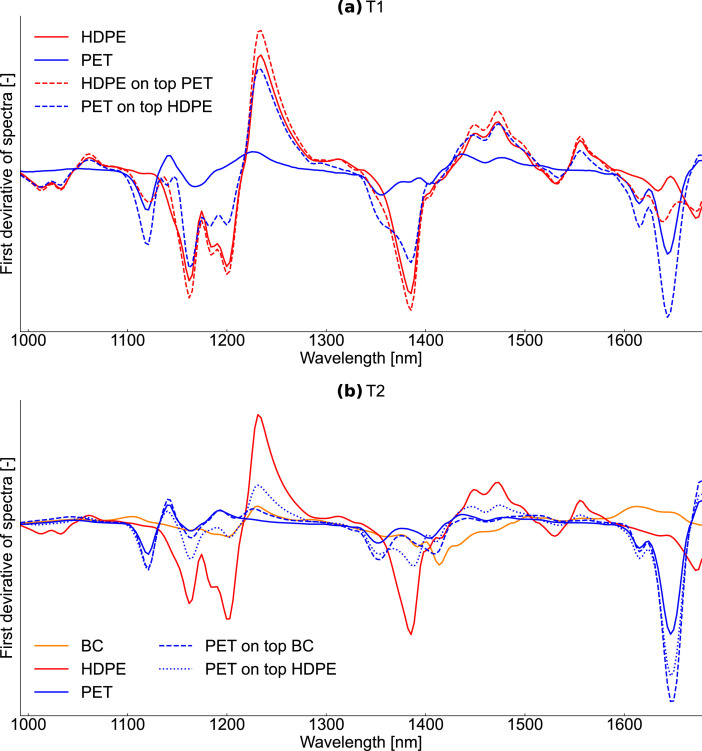
NIR reference spectra used for NIR classification at test series T1 and T2, cf. [1].

For defining NIR reference spectra shown in Fig. 5, representative regions of interest were selected for each material class. For T1, spectra were selected from the center of the plastic flakes to avoid edge effects [18,19]. Accordingly, reference spectra of non-sleeved and non-labeled parts of the LWP samples were selected for T2. Additionally, overlays of transparent materials on top of other materials were added as reference spectra to avoid systematic misclassifications due to mixed NIR spectra in the case of transparency (e.g., a PET bottle on top of a HDPE bottle is classified as PET), cf. [16].

#### 3.3.3 Export of False-Color Image

After each trial, the recorded false-color images of the “raw” section of this dataset were directly exported as “.bmp”-files. No further data processing was applied to the raw false-color images.

### 3.4 Data Preprocessing

The data preprocessing described below is intended to simplify subsequent data processing of users. However, users may also use the “raw” false-color images which are not affected by the data preprocessing. The described data preprocessing steps can be executed using the “preprocess.ipynb” Jupyter notebook included in the NIR-MFCO dataset. The preprocessing steps aim to remove irrelevant data (noise at the horizontal borders of the image and recordings of empty conveyor belt section at the beginning and end of each trial [white space]) and spatially calibrate the false-color images to ensure measurements independent of the particle orientation.

#### 3.4.1 Horizontal Cropping

During the false-color image recording, disturbances can occur at the edge of the conveyor belt (e.g., entangled particles at the edge of the conveyor belt). Therefore, horizontal cropping is applied as a first step in the data preprocessing to cut off these disturbances. Per default, 15 px are cropped off from the left and right border of each false-color image.

#### 3.4.2 White Space Removal

For the removal of white space, the false-color image is first segmented into a binary image, with the material areas representing the foreground (“True”) and the conveyor belt representing the background (“False”). Second, small image noise (e.g., dust particles that are classified as material) is removed by area-opening with an area threshold of 40 px. Third, the first and last continuous image lines without material pixels are vertically cropped off.

#### 3.4.3 Spatial Calibration

Each image is then spatially calibrated to ensure the same spatial resolution in *x*- (conveyor direction) and *y*-direction (“square pixels”) by resizing the image in conveying direction. The resizing factor depends on the frame rate and resolution (#pixels per line) of the NIR sensor as well as speed and width of the used conveyor belt. For spatial calibration, we recorded multiple circle calibration targets (T1: *d* = 60 mm, T2: *d* = 270 mm; cf. folder “_calib” in the raw folder of the dataset) and determined the mean bounding box dimension in *x*- and *y*-direction (${\bar{BB}}_{x}$, ${\bar{BB}}_{y}$). We then use these bounding box dimensions to determine a resize factor RF according to Eq. (3) and rescale the image by using the function *transform.rescale()* from *scikit-image* [20].

(3) $$RF=\frac{{\bar{BB}}_{y}}{{\bar{BB}}_{x}}$$

## Ethics Statement

This study does not involve experiments on humans or animals.

## CRediT authorship contribution statement

**Nils Kroell:** Conceptualization, Methodology, Software, Validation, Investigation, Data curation, Writing – original draft, Writing – review & editing, Visualization, Supervision, Project administration, Funding acquisition. **Xiaozheng Chen:** Conceptualization, Methodology, Validation, Writing – review & editing, Visualization, Funding acquisition. **Abtin Maghmoumi:** Software, Data curation, Writing – review & editing, Visualization. **Julius Lorenzo:** Investigation, Data curation, Writing – review & editing. **Matthias Schlaak:** Investigation, Writing – review & editing. **Christian Nordmann:** Resources, Writing – review & editing, Funding acquisition. **Bastian Küppers:** Conceptualization, Methodology, Writing – review & editing. **Eric Thor:** Writing – review & editing, Visualization. **Kathrin Greiff:** Supervision, Writing – review & editing, Funding acquisition.

## Declaration of Competing Interest

The authors declare that they have no known competing financial interests or personal relationships that could have appeared to influence the work reported in this paper.

## Data Availability

NIR-MFCO dataset: Near-infrared-based false-color images of post-consumer plastics at different material flow compositions and material flow presentations (Original data) (Zenodo).

## Appendix Supplementary materials

Image, application 1

Image, application 2

Image, application 3

Image, application 4

Image, application 5
